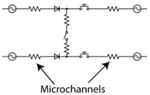# A Programmable Microfluidic Platform to Monitor Calcium Dynamics in Microglia during Inflammation

**DOI:** 10.21203/rs.3.rs-3750595/v1

**Published:** 2023-12-28

**Authors:** Adam Shebindu, Durga Kaveti, Linda Umutoni, Gia Kirk, Michael D. Burton, Caroline N. Jones

**Affiliations:** 1 Department of Bioengineering, University of Texas at Dallas, Richardson, TX 75080, USA; 2 Department of Biomedical Engineering, UT Southwestern Medical Center, Dallas, TX, 75390; 3 Department of Neuroscience, University of Texas at Dallas, Richardson, TX 75080, USA

## Abstract

Calcium dynamics significantly influence microglial cell immune responses, regulating activation, migration, phagocytosis, and cytokine release. Understanding microglial calcium signaling is vital for insights into central nervous system immune responses and their impact on neuroinflammation. We introduce a calcium monitoring micro-total analysis system (CAM-μTAS) for quantifying calcium dynamics in microglia (BV2 cells) within defined cytokine microenvironments. The CAM-μTAS leverages the high efficiency pumping capabilities of programmable pneumatically actuated lifting gate microvalve arrays and the flow blocking capabilities of the Quake valve to deliver a cytokine treatment to microglia through a concentration gradient, therefore, biomimicking microglia response to neuroinflammation. Lifting gate microvalves precisely transfer a calcium indicator and culture medium to microglia cells, while the Quake valve controls the cytokine gradient. In addition, a method is presented for the fabrication of the device to incorporate the two valve systems. By automating the sample handling and cell culture using the lifting gate valves, we could perform media changes in 1.5 seconds. BV2 calcium transient latency to peak reveals location-dependent microglia activation based on cytokine and ATP gradients, contrasting non-gradient-based widely used perfusion systems. This device streamlines cell culture and quantitative calcium analysis, addressing limitations of existing perfusion systems in terms of sample size, setup time, and biomimicry. By harnessing advancements in microsystem technology to quantify calcium dynamics, we can construct simplified human models of neurological disorders, unravel the intricate mechanisms of cell-cell signaling, and conduct robust evaluations of novel therapeutics.

## Introduction

Microglia are central nervous system (CNS)-specific immune cells that respond to both immunological^1^ and neuronal^2, 3^ signals. They are a subset of glia cells, which play an important role in maintaining homeostasis in the CNS^4^. Abnormal calcium signaling in glia cells has been associated with neuroinflammation^5^ which underlies several neurodegenerative diseases, including Alzheimer’s disease^6^ , Parkinson’s disease^7^, multiple sclerosis^8^, pain^9^, and sepsis^10^. Recent studies in mice have demonstrated the response of microglia to neuronal activity through spontaneous calcium dynamics^2, 11^. Moreover, calcium signaling is involved in mediating intercellular communication among microglia, as well as between microglia and other cell types, such as neurons and astrocytes during microglia activation^2, 12^. Therefore, studying calcium dynamics aids in unraveling the dynamics of these communication networks and their impact on brain function and is valuable for identifying novel therapeutic strategies and assessing the efficacy of drug interventions.

**Figure 1.**
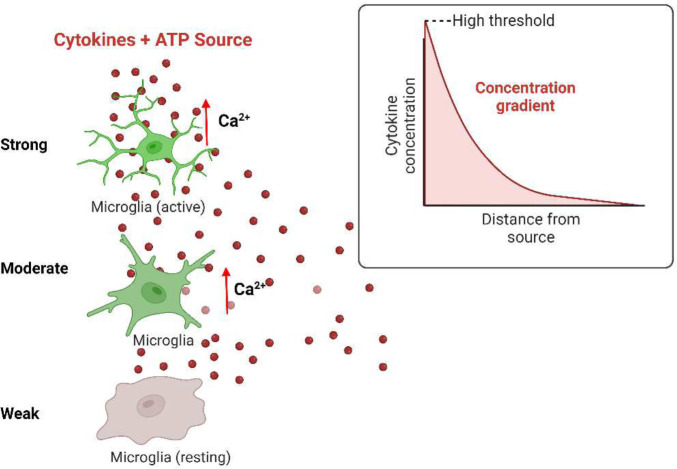
Location dependent microglia response to cytokine gradients. *In vivo,* microglia respond to neuronal (ATP) and immune stimuli (cytokines) through a concentration gradient. Microglia closer to the area of inflammation are activated first as the signal propagates across the CNS.

Current *in-vitro* calcium dynamics studies are done using a perfusion system that takes hours to set-up, use a high volume of reagents, requires millions of cells making it challenging to study human cells, and delivers treatment to cells through a uniform concentration of inflammatory signals^11, 13^. However, microglia sense and respond to inflammation in the human CNS through a concentration gradient of inflammatory signals such as cytokines, chemokines, or adenosine triphosphate (ATP)^14, 15^. Therefore, creating a concentration gradient during the delivery of a treatment to the cells is biomimetic, which increases the effectiveness of imaging calcium dynamics^12, 16, 17^(Fig. 1). The requirement to generate a gradient strengthens the demand for new microfluidics biomimetic tools that are efficient in terms of set-up time, reduction in cell sample size, and delivery of treatments to the cells.

Microfluidic systems are miniaturized and can be automated to deliver treatment to cells and generate a concentration gradient^17^. These systems have recently been used for calcium imaging in different *in-vitro* models including osteoblasts^18^, neuronal communication^19–21^, and astrocyte activation^22^. Additionally, Chokshi et al., introduced an automated microfluidic technology for the *in-vivo* study of calcium dynamics in *Caenorhabditis elegans*. This device enabled the processing of thousands of worms by immobilizing them, delivering a chemical odor to their nose touch, and recording the calcium transient data from single neurons while eliminating manual interactions with the device^23^. However, the presented technologies have been limited by either the lack of automation during the treatment delivery or creation of gradients to make the system biomimetic.

During the last two decades, microfluidic systems have been automated by incorporating fluidic control mechanism including, electrowetting^24^, acoustic^25^, and pneumatic actuations^26–29^. Among these actuation mechanisms, pneumatically actuated microvalves and micropumps have gained prominence due to their ease of fabrication, accuracy in volume control, high scalability, and simple integration with downstream systems. Microvalves and micropumps have been incorporated into microfluidic devices for fluidic control in several applications including; pathogen biosensing^30–32^, molecular preconcentration^33^, immunoassays^34^, and high throughput cellular analysis^35, 36^. Pneumatically actuated microvalves are divided into Quake valves and lifting-gate valves which are used separately based on the application. Lifting-gate valves are highly efficient with pumping efficiencies reported of up to 86%^27^, whereas the Quake valve uses a mechanical force to pinch a fluidic membrane and interrupt the flow^37, 38^. The Quake valve can be used as a blocking valve to create flow separation in channels of a microfluidic device^39, 40^. Both valve types use a monolithic, flexible PDMS membrane for actuation. However, they have several limitations. For instance, lifting gate valves produce backflow during actuation, and quake valves have poor resolution for flow metering. Table 1 summarizes the advantges and limitations of these valve systems. Despite offering the ability to pump efficiently while blocking fluid flow at specific positions, no system combining Quake valves and lifting-gate valves has been introduced.

We present a calcium monitoring micro-total analysis system (CAM-μTAS) that combines both the Quake and lifting gate valves. The integration of the pumping capabilities of lifting gate microvalves with the flow interrupting capabilities of the Quake valve enables quantitative analysis of calcium dynamics in microglial cells after exposure to a gradient of cytokines with temporal control. We validated this system and successfully automated calcium imaging of microglia during a dosed treatment of an “inflammatory soup” consisting of interleukin-1β, (IL-1β), interleukin-6 (IL-6), tumor necrosis factor-α (TNF-α), and adenosine triphosphate (ATP) because these pro-inflammatory cytokines are up regulated at the site of inflammation in the CNS^43^. This system automatically changed cell culture media with high efficiency, prepared cells for calcium imaging by on-chip calcium indicator incubation and exposed the cells to a concentration gradient of IL-1β, IL-6, TNF-α, and ATP. The CAM-μTAS also includes a pneumatically controlled microfluidic flow rectifier that enables zero backflow in all operations using the principle of an electrical transistor^44^. Eliminating backflow and ensuring steady flow allows the stabilization of cells during continuous pumping cycles of the lifting gate valves. As a result, the developed CAM-μTAS can be utilized in different cell models for calcium imaging.

## Materials and Methods

### Materials and equipment

The CAM-μTAS was fabricated by photolithography followed by soft lithography. To create the mold, we used SU-8 50 (Kayaku Advanced Materials, Westborough, MA), a negative photoresist, and AZ-12XT-20PL-10 (Microchemical GmbH, Germany), a positive photoresist, and silicon wafers (University Wafer, Inc., Boston, MA). For soft lithography, we used polydimethylsiloxane (PDMS) (Dow Corning, Midland, MI). To control the microvalve system, a control system was made using a series of SMC 3 solenoid valves (Steven Engineering, San Franscisco, CA), ULN2803 switch boards (Tempero Systems, Southport, Australia), and data acquisition (DAQs) devices (National Instruments, Austin, TX). The DAQs were connected to a laptop and controlled using a custom-made NI-LabVIEW program. All images were acquired by an inverted microscope (Nikon Ti-E, NY) equipped with a CCD-camera (EMCCD, Andor).

### Cell culture

To perform calcium dynamics on chip, we used BV2 cells- a C57BL/6 murine microglia cell line gifted by Malu G. Tansey (The University of Florida, Gainesville, FL). Upon receival, BV2 cells were grown in a medium constituted by 41.5% high glucose Dulbecco’s Modified Eagle’s Medium (DMEM, Gibco, Waltham, MA), 41.5% Opti-MEM (Gibco, Waltham, MA), 15% fetal bovine serum (FBS, Gibco, Waltham, MA), and 2% penicillin streptomycin (Gibco, Waltham, MA). BV2 cells were seeded in 25 cm^2^ flasks at density of 2×10^5^ cells/mL and placed in a humidified atmosphere incubator containing 5% CO_2_ at 37°C. The cell culture media was exchanged as needed (minimally twice a week) to supplement the cell line with fresh nutrients.

### Calcium imaging using a perfusion system.

Calcium imaging was performed as previously described by Li et. al^13^. Briefly, BV-2 Cells were plated on 0.1% poly-L-lysine coated glass bottom single well plates at a 2×10^6^ density and placed in a humidified atmosphere incubator containing 5% CO_2_ at 37°C overnight. The next day, cells were incubated with calbryte 520-AM at 37°C for 30 minutes. Following the incubation, cells were washed with an aqueous normal bath comprised of the following: 13.5 mM NaCl, 5 mM KCl, 10 mM HEPES, 2 mM CaCl_2_, 1 mM MgCl_2_, 10 mM glucose, titrated to pH 7.4±0.5. A custom-made perfusion system was used to deliver an inflammatory soup constituted of IL-1β [10 ng/mL] (R&D System, Minneapolis, MN), IL-6 [50 ng/mL] (R&D System, Minneapolis, MN), TNF-α [100 ng/mL] (R&D System, Minneapolis, MN), and ATP [200 μM] (Sigma-aldrich, St. Louis, MO), at a flow rate of 500 μL per second. While perfusing, calcium dynamics images were recorded using an Olympus IX73 inverted microscope (40X magnification, 100 ms exposure time) as shown in **SI Fig. 2A**.

### Fabrication of the calcium monitoring micro-total analysis system (CAM-μTAS)

**Figure 2.**
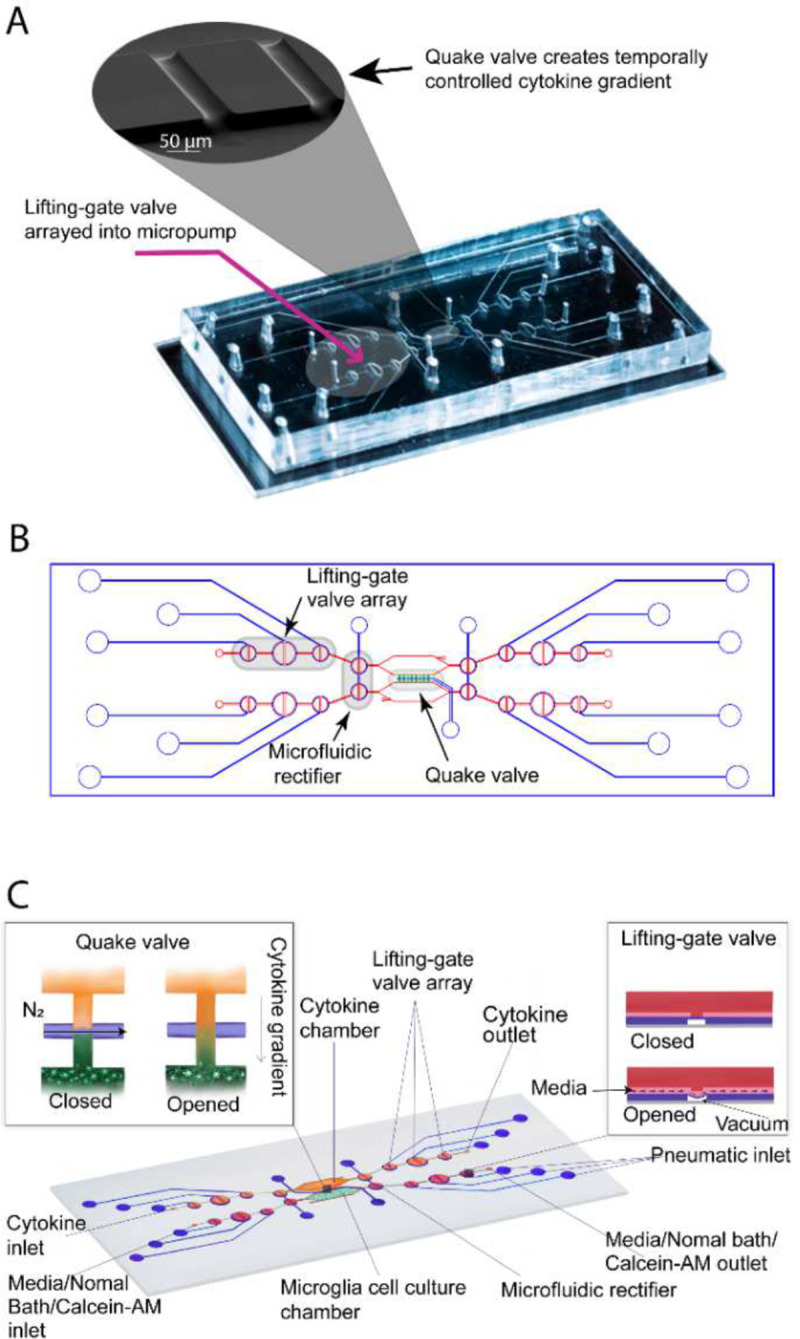
Calcium monitoring micro-total analysis system (CAM-μTAS) to quantify calcium dynamics in microglial cells. **A)** A fabricated CAM-μTAS including the lifting gate valve and a scan electron microscopy image of the Quake valve. **B)** CAD design of the CAM-μTAS, including lifting gate valves, microfluidic rectifiers and a quake valve. **C)** A feature illustration of the CAM-μTAS. The device combines a series of lifting gate valves into a micropump to automatically and precisely meter cell culture media changes, load calbryte 520-Am, and eliminate back flow. The CAM-μTAS leverages the flow blocking capability of the Quake valve to generate a concentration gradient, which enables a biomimetic deliver of inflammatory signals to the cells.

The CAM-μTAS illustrated in Fig. 2 consisted of a microfluidic layer which included the microglia cell culture and cytokine chambers, a pneumatic control layer, and a featureless glass wafer. The pneumatic layer was fabricated using standard photolithography techniques with SU-8 50 to achieve a feature height of 80 μm. A multilayer photolithography technique was used to fabricate the fluidic layer and obtain 40 μm feature height for the lifting gate valves and the chambers, and 7 μm for the Quake valves. First, SU-8 50 was spin-coated on a silicon wafer at 3000 rpm for the fabrication of the lifting gate valves and the chambers. To fabricate the Quake valve after developing the SU-8, the wafer was primed with hexamethyldisilazane (HMDS) for adhesion promotion, then spin-coated with AZ-12XT-20PL-10, a positive photoresist at 3500 rpm. The photoresist was cured for 3 minutes at 115°C then exposed to UV at a dose of 450 mJ/cm^2^ before baking it at 100°C for 1 minute and developing it by submersion in AZ 300MIF for 3 minutes. The Quake valve requires semi-circular channels for operation^37^. To achieve this, the wafer was placed on a hot plate at 135°C to allow the reflow of AZ-12XT-20PL-10. This process is summarized in **SI Fig. 1**. After coating both the fluidic and pneumatic layers with parylene-C (Specialty Coating Inc.) as an anti-sticking agent, a 10:1 ratio of PDMS (base material: curing agent) was poured onto the molds to obtain replicas. For the pneumatic layer, uncured PDMS was spin-coated on the mold at 915 rpm to obtain a 100 μm thick PDMS membrane and for the fluidic layer, PDMS was poured on the mold. Both were cured at 65 °C in an oven overnight. Holes were punched in the PDMS replica with a biopsy punch for fluidic inlets and outlets, and then the fluidic layer was aligned and permanently bonded to the pneumatic layer using air plasma activation (Plasma Etch Inc.) at 350 mTorr chamber pressure and 90 W power for 3 minutes to form the CAM-μTAS. Lastly, holes were punched in the CAM-μTAS for pneumatic connection wells and the CAM-μTAS was bonded to a single well glass bottom plate (MatTek) after air plasma activation and placed on a hot plate at 80°C for 15 minutes.

### Chip operation and flow optimization around cells

The CAM-μTAS was optimized for flow rate by applying vacuum for opening at −85 kPa and varying the closing pressures at 15 kPa, 30 kPa, and 45 kPa to the pneumatic lines by the solenoid controller. The volume pumped per cycle is a function of closing pressure and actuation time^27^. **SI video 1** summarizes the pumping mechanism used for the device operation. After flow optimization, a closing pressure of 45 kPa and an actuation time of 500 milliseconds were selected because this condition yielded the highest flowrate (355 nL/cycle) at the lowest actuation time (500 ms). Prior to operating the CAM-μTAS, all microvalves were opened and the chambers were filled with 0.1% poly-L-Lysine for 2 hours to functionalize the PDMS surface and improve cell adhesion. After 2 hours, poly-L-Lysine was washed 3 times by pumping in sterile DI water before adding cell culture media. Microglia (BV2) cells were added to the microglia cell culture chamber at a 2×10^6^ cells/mL, and the chip was removed from the controller and placed in a humidified incubator with a 5% CO_2_ atmosphere at 37°C for 24 hours to allow the cells to adhere. The next day, the chip was installed on the controller and 25 cycles were pumped to test the rate of detachment of cells from the surface of the chamber. This process was repeated under different surface treatments including 0.1 % poly-L-Lysine, and 0.1 % poly-L-Lysine mixed with fibronectin [11 μg/mL]. While pumping, time lapse images were taken at an interval of 1 second for three minutes at 20X magnification to observe the rate of cell detachment. This rate was quantified in terms of the percentage of cells removed from the field of view after the 25 pumping cycles.

### Automated calcium indicator incubation

To perform calcium imaging, cells are incubated with calbryte 520-AM (AAT Bioquest, Pleasanton, California). A 4 μM solution of calbryte 520-AM was prepared in an aqueous normal bath comprised of the following: 13.5 mM NaCl, 5 mM KCl, 10 mM HEPES, 2 mM CaCl_2_, 1 mM MgCl_2_, 10 mM glucose, titrated to pH 7.4±0.5. The normal bath was adjusted to an osmotic pressure of 300±5 mOsm. Three cycles of calbryte 520-AM were pumped into the microglia cell culture chamber and the cells were incubated at 37°C for 30 minutes. Time-lapse images were taken using the Nikon Ti-2 fluorescent microscope with a biochamber (FITC, 20X magnification).

### On-chip microglia calcium imaging

To demonstrate the utility of the CAM-μTAS, the microglia cell culture chamber was primed with 0.1% poly-L-lysine for 2 hours at room temperature. After washing the poly-L-lysine by pumping 3 cycles of sterile DI water, 400 nL of BV2 cells suspended in media at a density of 1.98×10^6^ cells/mL (~800 cells) were loaded into the microglia cell culture chamber. The CAM-μTAS was removed from the controller and placed in a humidified incubator with 95% air and 5% CO_2_ atmosphere at 37°C for 24 hours to allow the BV2 cells to adhere. The next day, the media was replaced by a normal bath solution described above. Calbryte 520-AM was pumped into the system and then washed out after 30 minutes. The CAM-μTAS was placed on the Nikon Ti-2 fluorescent microscope mounted with an incubator at 37°C. In the adjacent chamber, a solution diluted in normal bath and containing IL-1β [10 ng/mL], IL-6 [50 ng/mL], TNF-α [100 ng/mL], and ATP [200 μM], was added while the Quake valve was closed. Once the treatment was in place and the cells were fluorescent with calbryte 520-AM, the Quake valve was opened, and a gradient was allowed to form for 10 minutes. We repeated this experiment in 3 devices.

### Statistical Analysis

Statistical analysis was performed using Prim-GraphPad 8. Data from at least three experiments were analyzed and presented as mean ± standard deviation. For a given experiment, each condition was tested in triplicate. One-way ANOVA with a statistical significance level of 0.05 was used to determine the difference in chip surface treatment for cell adhesion.

### Image Analyses

Image analyses were performed using Nikon Element AR 5.41.02 and data were plotted using Prism-GraphPad (San Diego, California). For calcium dynamics, data are expressed in ΔF/F ratio where F is the intensity at time t=0 seconds while ΔF is the difference in intensity at time t=0 seconds, and at the time of cell response.

## Results

### Combining lifting gate valves and Quake valve to create the CAM-μTAS

The lifting gate valves, and the Quake valve have distinct fabrication techniques which require negative and positive photoresist, respectively. To incorporate the two valve systems on the same device, the fluidic layer was fabricated using a photomask printed with the lifting gate valve circuit and another photomask printed with the Quake valve. During fabrication, the lifting gate valves are fabricated first because positive photoresists are not compatible with SU-8 developers. The Quake valve layer is aligned to the lifting gate circuit as shown in **SI Fig. 1**. The components of the circuit are summarized in Table 2. To operate the Quake valve, fluidic channels must have a semi-circular roof to allow the membrane to deflect. We fabricated the Quake valve channels with a height of 7 μm and a width of 50 μm to eliminate the dependence of actuation pressure on the depth of the fluidic channel (**SI Fig. 1**). The fabricated device was mounted to a control system as illustrated in **SI Fig. 2B** and used the push-up method for pneumatic actuation^38^. Mounted to a pneumatically controlled manifold, the lifting gate valve system operates as an alternating flow generator while the Quake valve operates as a programmed switch (Table 2).

**Figure 3.**
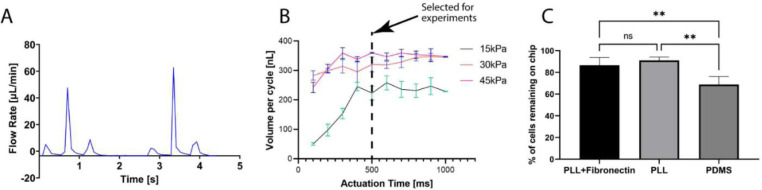
Backflow elimination, and flow optimization for cell adhesion in different surface treatments. **A)** volumetric profile of the flow rate recorded during actuation which shows complete elimination of backflow during actuation. **B)** Volume pumped per cycle as a function of actuation time and closing pressure of the microvalve. The valve actuation vacuum was −85 kPa, and the pressure varied from 0 to 45 kPa. For the experiment an actuation time of 500ms was selected at 45 kPa closing pressure. **C)** The percentage of cells retained on the device under 45kPa valve closing pressure and 500ms actuation time for different PDMS surface treatment. (Ordinary one-way ANOVA; n=4, *p<0.05, no significant difference).

Lifting gate valves have been reported to generate backflow during continuous pumping processes^44^. This continuous instability caused by backflow in the microglia cell chamber can lead to cell detachment or death. To address this, the CAM-μTAS included a pneumatically controlled microfluidic flow rectifier to regulate and reduce the flow rate while pumping around cells while also playing the role of a diode to eliminate backflow (**SI Fig. 3**). Therefore, prior to the operation of the CAM-μTAS we evaluated the effectiveness of the microfluidic flow rectifier by recording the flow profile during actuation. As seen in Fig. 3A, the CAM-μTAS eliminated backflow in the cytokine and microglia cell culture chambers.

### Flow characterization and cell retention

The CAM-μTAS consisted of twelve lifting gate valves, four flow rectifying valves, and one Quake valve to allow access to two fluid inlets and two outlets to the system. The lifting gate valves were arranged in groups of three to include two gate valves with a diameter of 1 mm and one central pumping valve with a diameter of 1.5 mm. The valves were connected by 50 μm wide channels leading to the 4×2 mm microglia cell culture and cytokine chambers. The 1.5 mm diameter and the 80 μm pneumatic height of the pumping valve were selected to ensure a maximum deflection of the pneumatic membrane during actuation. The arrangement of the lifting-gate valves in series of three effectively created micropumps, which were used to transfer cell culture medium, normal bath solutions, calcium indicators to the microglia cell culture chamber, and cytokines to the cytokine chamber.

Pumping flow rate was measured and expressed in terms of volume dispensed per pumping cycle as a function of closing pressure and actuation time. As previously observed^27^, at fixed actuation time, the volume flow rate increased as a function of closing pressure. However, the pumping valve has a maximum membrane deflection height. Therefore, as the actuation time increases, the valve reaches its maximum holding capacity (Fig. 3B). The maximum volume pumped per cycle was reached at 45 kPa closing pressure and 500 ms actuation time. This condition was selected for all the downstream experiments. Under this condition, the lifting gate valve system yielded a pumping efficiency of 73.3% calculated from the theoretical pumping valve capacity.

Microglia are adherent cells that attach to the surface on which they are seeded^45^. However, the adhesion of cells on PDMS surface has been shown to be weak in general^46^. Therefore, the surface requires functionalization to ensure that cells are retained to the surface. We used two different surface treatments, 0.1% poly-L-lysine and 0.1% poly-L-lysine reinforced with fibronectin [11 μg/mL] and tested the cell retention rate against the flow rate at 45 kPa and 500 ms actuation time. We found no significant difference between treating the surface with only poly-L-lysine and poly-L-lysine reinforced with fibronectin (Fig. 3C). Therefore, poly-L-lysine was selected as the surface treatment agent for all the subsequent experiments because it had the lowest average cell detachment.

### Calcium dynamics of microglia on the CAM-μTAS

**Figure 4.**
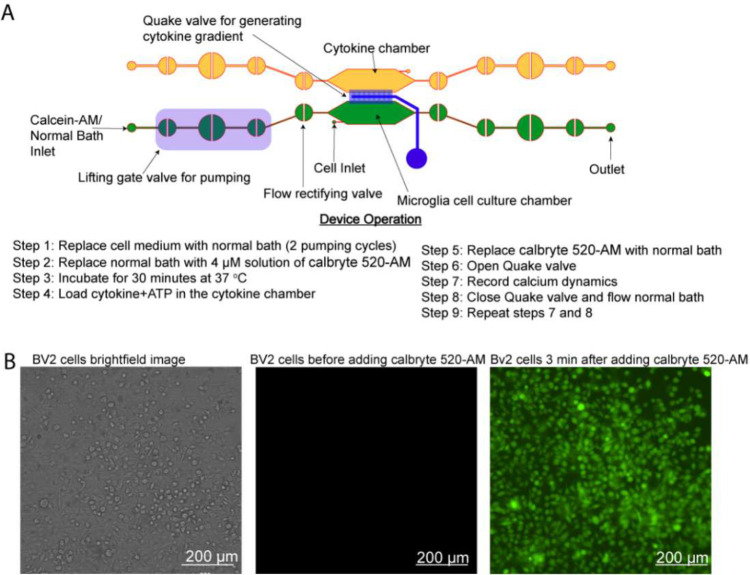
Device operation and calcium Indicator loading. **A)** The process of actuating the lifting gate valves in series to create a micropump following a continuous flow regime in order to load calbryte 520-AM/normal bath/cell medium to the microglia cell culture chamber and cytokines+ATP to the cytokine chamber. **B)** Microglia cells before and after calbryte 520-AM incubation. Miniaturization accelerated the incubation process allowing calbryte 520-AM to penetrate the cells in 30 minutes. (20X magnification).

**Figure 5.**
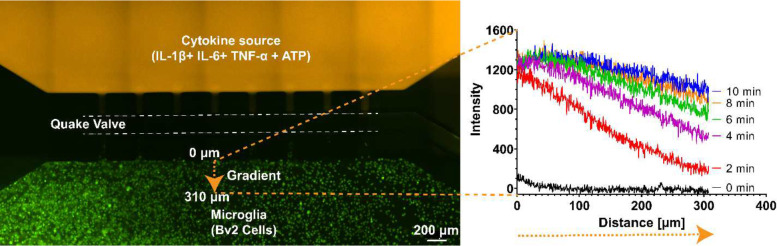
Controlled cytokine gradient. A concentration gradient was generated to deliver pro-inflammatory cytokines (IL-1β [10ng/mL], IL-6 [10ng/mL], TNF-α [5ng/mL], and ATP [200μM]) to the cells. As seen in the above fluorescence plot, the decreasing concentration of the signal from the signal chamber to the cell culture chamber makes the CAM-μTAS biomimetic. Dextran was added to the cytokine solution for fluorescent imaging purposes.

The Quake valve incorporated in the CAM- μTAS played two roles. On one hand it allows the compartmentalization of the microglia cell culture chamber and the cytokine chamber. On the other hand, it controlled the delivery of treatments to cells through a concentration gradient. The Quake valve enabled us to model inflammation by creating a user-controlled gradient of inflammatory signals toward the microglial cell culture chamber. These signals included IL-1β [10 ng/mL], IL-6 [50 ng/mL], TNF-α [100 ng/mL], and ATP [200 μM] ^47^. The signal solution was pumped into the cytokine chamber through the automated lifting gate valve system (Fig. 4A). Simultaneously, calbryte 520-AM was pumped into the microglia cell culture chamber as a calcium indicator. After 30 minutes, cells were fluorescent as shown in Fig. 4B. Upon opening the Quake valve the gradients reached the BV2 cells. Fig. 5 illustrates the progression of the gradient over time as recorded on NI element AR using the TRITC channel of the Nikon-Ti fluorescent microscope. As the gradient formed and the cytokine + ATP treatment was being delivered to the cells, time-lapse images were recorded at 1 second intervals for 10 minutes starting from the time when the Quake valve was opened.

**Figure 6.**
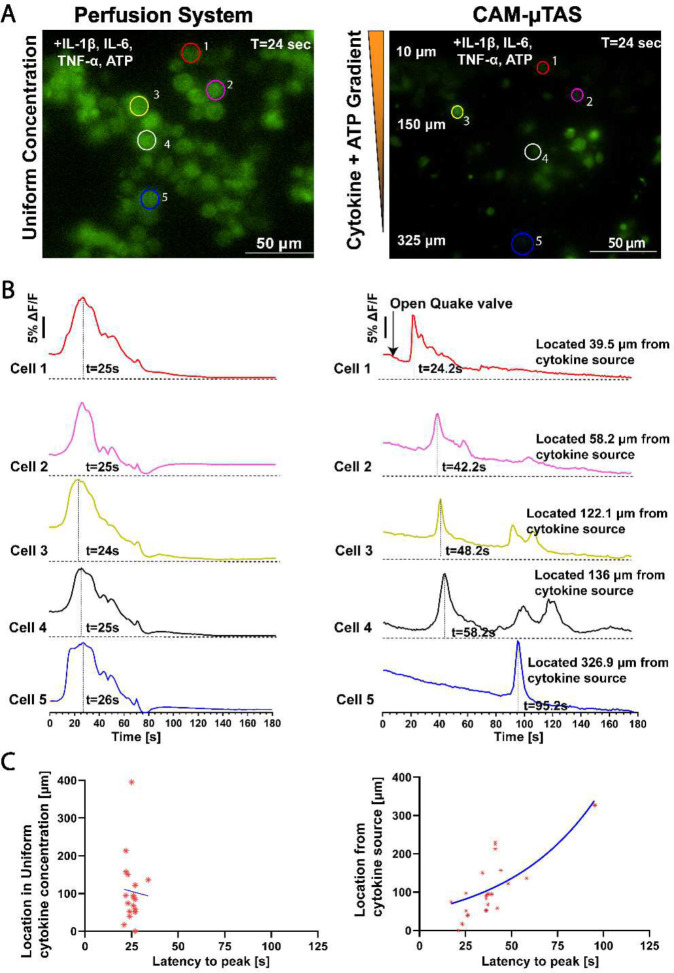
Pro-inflammatory cytokines (IL-1β, IL-6, TNF-α, and ATP) induced a [Ca2+] transient elevation in microglia. A) 40x Fluorescent FITC calcium image of BV2 cells at the peak calcium transient intensity using the perfusion system and using the CAM-μTAS. B) Time series of the fluorescence intensities of responsive cells during the application of IL-1β [10ng/mL], IL-6 [10ng/mL], TNF-α [100ng/mL], and ATP [200μM] using the perfusion system and using the CAM-μTAS. On the CAM-μTAS, microglia response to the gradient is location dependent (the locations displayed are measured away from the cytokine source). C) Location vs. latency to peak of cells using the perfusion system and using the CAM-μTAS. There is no correlation between location and latency to peak in the perfusion system (r=−0.046), but the CAM-μTAS showed a strong correlation (r=0.76) (n=3 devices).

Using the CAM- μTAS we were able to quantify the calcium dynamics in single microglia and observed that 45% of sampled cells showed calcium transient activity (n=43) whereas using the perfusion system the number was 42% (n=54). Conventional cell calcium dynamics imaging uses a perfusion system, which operates with ON/OFF valves to deliver treatment to cells in a stepwise manner as shown in **SI Fig. 2C.** However, the CAM- μTAS delivers the treatment following a concentration gradient. In the traditional perfusion system, cells responded at the same time (25±2 seconds) after perfusing the well plate as shown in Fig. 6A&B. However, in the CAM- μTAS, cells responded following a concentration gradient of IL-1β, IL-6, TNF-α, and ATP. Cells closer (<10 μm) to the source started to respond 20±3 seconds after opening the Quake valve as shown in **SI Video 2**. As the treatment reached the cells downstream (10–325 μm) there was a location-dependent response with a positive correlation (r=0.76) as shown in Fig. 6C. However, in the perfusion system, all the responsive cells responded at a relatively fixed time with no location dependence (r=−0.048). As shown in Fig. 6B, we observed a 10-fold decrease in the intensity of calcium transient signal using the CAM- μTAS. This is because, unlike the perfusion system which delivers uniform high concentrations of IL-1β, IL-6, TNF-α, and ATP to cells as shown in **SI Fig. 2C**, the CAM- μTAS creates a gradient. Therefore, at the time of cell response, the concentrations of IL-1β, IL-6, TNF-α, and ATP are lower than the source concentrations as shown in Fig. 5.

## Discussion

Traditionally, intracellular calcium imaging has been studied using a perfusion system such as the one shown in **SI Fig. 2A**. Although this system is functional and offers high temporal resolution, it uses a high volume of reagents and a large sample size^13^. In brain science, it is often difficult to obtain such large sample sizes. Moreover, the perfusion system also fails to create a concentration gradient during the delivery of treatments which makes it less biomimetic. In microfluidics, however, cell calcium imaging is performed by using manually operated devices that lack a concentration gradient^48, 49^. We contribute to previous efforts by adding an automatically actuated pneumatic microvalve system which combines the lifting gate valves and a Quake valve to generate a concentration gradient as shown in Fig. 1 and setup in **SI Fig. 2B**. Lifting gate valves have been previously used for high throughput biochemical processing with reported efficiencies as high as 86%^27, 33^. To our knowledge, however, these valves have not been used to automate cell culture mainly because the actuation of lifting gate valves is followed by backflow which can cause cell death. The CAM-μTAS addresses this issue by incorporating a microfluidic flow rectifying valve^44^ which eliminates backflow at the inlet of the cell culture chamber as seen in **SI Fig. 3.** Adding automation to cell culture enabled us to accelerate sample handling steps including media changes, calcium indicator incubation, and delivery of treatment to cells. These steps normally take several minutes to complete. However, with the CAM-μTAS we achieved them in seconds. For example, to change the medium in the cell microglia cell culture chamber at a 500 ms actuation time (Fig. 3B) only took 1.5 seconds in total.

Incorporating Quake valves and lifting gate valves on the same device adds complexity to the fabrication process. However, replacing the Quake valve used in the CAM-μTAS with a series of lifting gate valves introduces a significant amount of dead volume which is not suitable for controlling the chemical gradient during the delivery of the treatment to the cells. Therefore, separating the microglia cell culture chamber and the cytokine chamber with a Quake valve not only creates a physical separation between the chambers, but also provides the formation of a user-controlled cytokine gradient. The user can actuate the valve at any time or program the actuation to deliver the treatment to cells or to stop the delivery of treatment to the cells without mechanically affecting the cells.

Microfluidics devices have gained prominence for CNS cell calcium imaging due to the reduction in sample size, high temporal resolution, and single cell resolution analysis^50^. The CAM-μTAS contributes to this effort by adding the spatial resolution factor to the analysis. During the administration of cytokine gradient to microglia, we observed that microglia respond to the treatment through a concentration gradient and several cells had more than one peak as shown in Fig. 6, **SI Fig. 4** and **SI video 2**. This result shows that as the concentration of cytokines increases, cell activity also increases as indicated by multiple calcium transient peaks. This observation was only possible due to the delivery of cytokines through a concentration gradient. However, using the perfusion system, we could only observe an instant calcium dynamic at the application of the treatment and no further response from microglia. We plan to further investigate the cells that showed more than one calcium peak as the cytokine concentration increased in the microglia cell culture chamber. However, we believe that it is due to the heterogeneity in the microglia population.

The CAM-μTAS successfully allowed the monitoring of calcium dynamics by introducing various innovative features. However, the current device does not offer control over the distribution of cells in the chamber. This limitation can be addressed by designing cell blocks in the chamber that can hold single cells^48^. Additionally, the stability of flow in the microglia cell culture chamber can be improved by applying an external pressure to the microfluidic flow rectifier as described by Bavil, et. al^44^. However, this technique comes at the cost of pumping efficiency.

The current device can be used for various applications including drug screening, cell-to-cell communication, and cell chemotaxis. For drug delivery, other brain cells can be cultured in the microglia cell culture chamber and drugs can be delivered to the cell through the Quake valve following a concentration gradient and a response can be measured in real time. For cell-to-cell communication, different cell types can be cultured in the two chambers and the Quake valve can be actuated to allow the cells to communicate. Finally, the CAM-μTAS can be used to quantify cell chemotaxis. Here, a chemoattractant can be added in the cytokine chamber and a gradient can be generated through the Quake valve to allow the cells to migrate following the chemoattractant gradient. In conclusion, the addition of automation to microsystems promises a new era of precision, speed, and consistency in understanding and manipulating human cellular behavior, opening the doors for advancements in personalized medicine, disease modeling, and drug discovery.

## Conclusion

We have developed a calcium monitoring micro-total analysis system (CAM-μTAS) enabling the measurement of calcium dynamics in microglia following a cytokine treatment. Equipped with an automated fluid pump, microglia cell culture and cytokine chamber separating Quake valve, this device enables programmed and metered fluidic manipulations with no human interaction. In this work, we have optimized and validated the CAM- μTAS for calcium imaging. We were able to quantify a location dependent calcium dynamics response of microglia to a cytokine gradient. The device leverages the strength of both the lifting gate microvalve arrays and the Quake valve for cell loading and fluid processing including media change, cell incubation with calcium indicator, gradient formation, and cytokine delivery to the cells. The developed device is fabricated using a combination of both positive and negative photoresists to allow the incorporation of the two different valve systems. The CAM-μTAS introduces an innovative method in the fabrication of microfluidic pneumatic control systems.

## Figures and Tables

**Table 1.** Comparison of pneumatically actuated microsystems.

Valve Type	Advantages	Limitations	Applications
**Lifting gate valve (Normally-closed, Actuate to open)**	High pumping efficiency Used in series to form micropumps.	Need for valve passivation to avoid them becoming permanently closed. Pumping backflow causes unsteady laminar flow.	Precise metering of reagents ^33^ Rapid biosensing and point-of-care diagnostics^30^
**Quake valve (Normally-opened, Actuate to close)**	Used to interrupt flow without causing unsteady laminar flow. Used to create a concentration gradient.	Poor resolution for flow metering The height and the width of the valve cannot be designed independently.	Immunoassays^41^ Cell sorting^42^
**System combination of Lifting gate and Quake valves**	Leveraged the pumping capabilities of normally closed valves for high efficiency fluidic manipulations. Uses normally opened valves to block flow at a targeted location to create isolated chambers. Allows the formation of a concentration gradient in channels.	Fabrication challenges due to non-compatibility of photoresists and developers for normally closed and normally opened valves.	Drug screening to cells Cell co-culture Cell migration to a gradient

**Table 2.** Microfluidic Circuit Components (Scale Bar: 1 mm)

Component	Image	Function	Circuit Symbol

Lifting gate valves Micropump	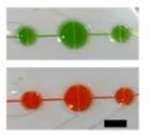	To pump reagents including cell culture media, normal bath, or calcium indicator to cells	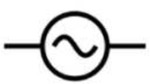
Quake Valve	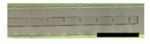	To create a chemica gradient during the delivery of cytokines cells	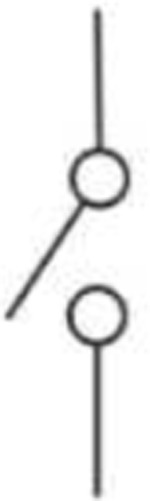
Microfluidic rectifier Diodic valve	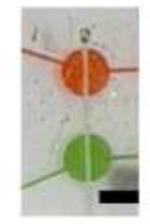	Ensure backflow elimination and stabilization of flow within the cell culture chamber	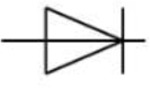
Gate Valve	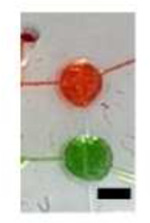	Allow separation of downstream pump from the cell culture and cytokine chambers	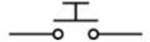
Cytokine and cell culture chambers	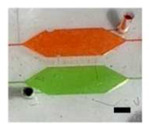	Storage of cytokines and cells	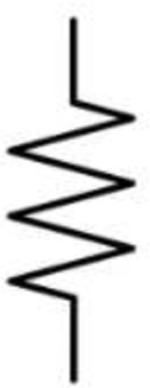
CAM-μTAS	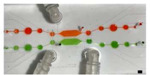	Fully automated microfluidic system to monitor calcium dynamics in microglia following a cytokines concentration gradient-based delivery